# COVID-19 and the taxation of professional athletes’ image rights

**DOI:** 10.1007/s40318-020-00180-w

**Published:** 2020-12-15

**Authors:** Sarah Carrick

**Affiliations:** Faculty of Arts and Humanities, University of Stirling, Stirling, Scotland, UK

**Keywords:** Athlete, Image, Rights, Taxation, HMRC, Footballers

## Abstract

The COVID-19 pandemic has raised and will continue to raise issues for sport for some time to come. In particular, the pressure put on athletes by politicians to take wage deductions and wage deferrals has caused controversy. This scrutiny of athletes’ tax affairs is not unusual, given the particular and somewhat constant media focus, coupled with HMRC’s ‘Football Compliance Project’ regarding footballer’s use of image rights companies to make tax deductions. This focus often presents footballers in an unflattering light. However, the purpose of this article is to demonstrate that the issues regarding athlete image rights are generally not due to overly sophisticated or overt actions by footballers and/or their agents but are the consequence of a convoluted system of taxation. Ultimately, HMRC guidance allows athletes to exploit an ‘image right’ to make tax savings. This ‘image right’ does not exist in law. Thus, this article will show that the current controversy surrounding footballers and their tax affairs is not a novel concept and that in the context of image rights athletes, clubs and agents have been forced to navigate a system of tax which is confusing, at best. In short, the issues surrounding footballers and image rights are due to the fact that ‘image rights’ are protected in one area of law yet do not exist in another.

The COVID-19 pandemic has and will continue to have an unprecedented and unknown economic impact around the world for some time to come. Stock markets have plummeted, those seeking unemployment benefit has risen at an exponential rate and the travel and hospitality markets have been decimated.^1^ Similarly, the entertainment industry, including sport, music and television production, has come to a halt.^2^ Amongst this chaos and economic uncertainty, professional footballers in particular have come under significant scrutiny, particularly from politicians, in the hope of pressuring them into taking wage cuts. This scrutiny was intensified by Premier League Clubs, including Liverpool and Tottenham, taking advantage of the Governments’ ‘Job Retention Scheme’ which allows employers to claim 80% of an employee’s wages by placing them on furlough.^3^ The clubs placed non-playing staff on this scheme, leaving the taxpayer to foot the bill, sparking a social media backlash from fans, players and the general public. Ex Liverpool star Jamie Carragher claimed he was “angry and embarrassed”^4^ whilst journalist and Good Morning Britain panellist Piers Morgan branded the move “astonishingly stupid” and caused Liverpool’s reputation “irreparable damage”.^5^ To put the public outrage in perspective, league leaders Liverpool made a £42 million pre-tax profit last season, from a £533 million revenue.^6^ Although both clubs have now reversed their decision to join the scheme, the controversy did not end there. Health Secretary Matt Hancock called on stars to “play their part” and take a 30% wage cut in the form of deductions and referrals,^7^ whilst Sadiq Khan, Mayor of London, claimed footballers have “the greatest shoulders to carry the greatest burden”.^8^ This political backlash against footballers was met with disapproval, with Wayne Rooney voicing his opinion that footballers had been made “scapegoats” and “easy targets”^9^ whilst Gary Lineker highlighted the absence of calls to those wealthier than footballers to help, including CEOs, bankers and millionaires.^10^ The PFA responded by stating that “the proposed 30% salary deduction over a 12-month period equates to over £500 m in wages and in tax contributions of over £200 m to the government, “What effect does this loss of earning to the government mean for this NHS?””^11^ Instead, footballers around the country launched the ‘#playerstogether’ initiative in which stars have donated to the NHS.^12^

However, in highlighting the undoubtedly extensive tax bill of professional footballers through the PAYE system, the PFA has also inadvertently drawn attention to the general tax affairs of the sports top stars, another area which has attracted political and media attention, as well as that of HMRC. In 2015, HMRC launched the ‘Football Compliance Project’, investigating possible tax evasions amongst clubs, agents and athletes themselves and as of January 2019, an additional tax revenue of £396 m had been recovered.^13^ It has been reported that HMRC is currently investigating 330 footballers, 80 agents and 50 clubs.^14^ These numbers are an increase from 173 footballers, 38 agents and 40 clubs in January 2019.^15^ Such attention from HMRC and the media often comes from the tax savings they make in respect of image rights payments. This raises the question as to whether taxation should operate on the basis, or at least in consideration of, commercial concepts and interests, or whether taxation should operate separately from such interests,^16^ reinforcing the argument for reform. As such, the current friction between footballers, politicians and the media will not result in new investigations given the ongoing nature of the compliance project, but what it has done is brought the tax affairs of footballers to the wider public attention once more and re-enforces the need for athletes, agents and accountants to understand the intricacies of footballers’ image rights in relation to taxation. However, in attempting to understand these issues, there must be an acknowledgement that the so-called tax avoidance culture of athletes is “in part the consequence of governmental attitudes to taxation and inadequacies in our tax system”.^17^ Thus, this paper will outline and explain these intricacies and critically analyse the key issue; *the fact that image rights are recognised in one specific area of law (tax) but not in any other context, illustrating that the controversy surrounding the taxation of footballers is generally not created by footballers or their advisors ‘going all out’ to avoid tax, but by a convoluted system of taxation which creates confusion for all involved and reinforces the argument and the need for a statutory image right.*

## Image rights challenges

Before dissecting the specifics of image rights and taxation, it is first necessary to highlight the tax authority’s consideration of image rights payments. The *Sports Club plc and others v Inspector of Taxes*^18^ litigation dates back to 2000 where although HMRC challenged the legality of image rights payments, clubs were essentially given the ‘green light’ in making such payments.^19^ HMRC launched an investigation into Arsenal Football Club in respect of their image rights payments to two of their players, David Platt and Dennis Bergkamp. Both players had offshore image rights companies with whom Arsenal had entered into agreements to allow them to exploit the image of both players in return for a fee. HMRC argued this fee was salaried remuneration and therefore subject to tax.^20^

Both players appealed against the decision which held that the payments were salary payments. The issue in contention regarded the application of s19 of the Income and Corporation Taxes Act 1988. The relevant part of Section 19(1) states that tax shall be charged in respect of any employment or emoluments therefrom, indicating that any payments stemming from employment are liable to income tax.^21^

The main issues were as follows: did the promotional and consultancy agreements have independent values? Were the payments for the promotional agreements simply a smokescreen in order to pay additional remuneration and were the payments under the agreements with the footballers’ emoluments from the employment?^22^

Accepting the players’ argument that image rights can be defined as ‘the ability to exploit their image in a commercial context in exchange for a fee’, the tribunal held the agreements were “genuine commercial agreements which the parties could seek to enforce”.^23^ In assessing whether these agreements had independent value, in light of the evidence that commercial organisations had been willing to pay substantial sums of money in order to secure the promotional services of these players, the agreements did indeed have independent value of their own.^24^

With regard to the argument that these payments were a smokescreen for additional remuneration, the court answered this question in the negative. The tribunal considered that given the process followed by the parties to adequately value the image rights and the substantial value of the players’ image rights before joining Arsenal, the payments could not be considered a smokescreen for additional remuneration.^25^

In reference to the final question as to whether the payments were emoluments from the employment contract, it was held:

“The promotional agreements and the consultancy agreement were contracts for full consideration and so would be excluded from tax under s19 for that reason alone. Also, we find the payments under those agreements were made in return for promotional rights and consultancy services respectively and were not made “in reference to” the playing of games which was the service rendered by each player by virtue of his player’s agreements with Sports. Neither were the payments under the promotional and consultancy agreements a reward paid by Sports for the services of the players; they were paid by Sports for the promotional rights and the consultancy services respectively.”^26^

By virtue of the ruling of *Sports Club*, *clubs have been able to use of image rights contracts legally in order to secure the promotional services of their star players, whilst utilising the tax benefits for clubs and players alike*. These structures can be considered beneficial and as Freedman explains, “naturally, taxpayers will do what they can to minimise their tax bills”.^27^ However, there has been controversy in recent years as to whether many agreements can be considered genuine. During the 2000s many clubs, across a number of sports, entered into image rights contracts with players who lacked the commercial prowess to attract the kind of sums they were being paid,^28^ alerting the revenue to the possible disguised remuneration. In 2006, HMRC launched a review of image rights payments in Rugby Union where a “cap of 15% of remuneration payable for image rights exploitation was agreed in Rugby Union”,^29^ whilst in 2009, a number of Premier League Clubs were informed it would be examining image rights agreements between 2005 and 2008.^30^ Although HMRC formally denies this,^31^ it has been claimed that Premier League Clubs were offered a “two cap” solution. The ‘Club cap’ “states that clubs can make maximum total image rights payments to all image rights companies of 15% of commercial income”,^32^ whilst the player cap sits at 20% of the total of salary payments made to any one player in the tax year.^33^ In spite of these apparent agreements, however, HMRC’s Football Compliance Project is indicative of continuing issues of non-compliance, but as this article will illustrate, this is due to the non-existence of an image right in law and lack of guidance as to what ‘image’ constitutes. This is discussed in further detail as follows. Although it is important to acknowledge the role of *Sports Club* in legitimising image rights payments, it is also important to acknowledge that the case dates back twenty years and the recent Supreme Court decision *in RFC* 2012*Plc (in liquidation) (formerly The Rangers Football Club Plc) v Advocate General for Scotland*^34^ is authority for the preposition that any payment made to an employee for services rendered is taxable as earnings, even if it is paid to a third party.^35^ Thus, were *Sports Club* to be decided today, it is a plausible argument that clubs’ payments to a third party, in this case an image rights company, could be regarded as employment income by virtue of the precedent set down in *Rangers*. This argument may be a useful weapon for HMRC in utilising the Football Compliance Project.^36^

In the first decision specific to image rights since *Sports Club*, the 2019 decision of the First Tier Tribunal in *Hull City (AFC) Tigers Limited v The Commissioners for HMRC*^37^ strengthens the above argument and provides a useful illustration of the tax tribunals’ approach to image rights payments in respect of professional football.^38^ The case concerned Hull City FC who paid player Giovanni Gomez’s overseas image rights company £440,800 between December 2008 and July 2010.^39^ The question was whether these payments could be categorised as earnings subject to income tax.^40^ Having taken “a realistic view of the payments and considering their substance and not just their form”,^41^ it was held that, in contrast to *Sports Club*, the payments were indeed remuneration and not image rights payments. Of particular use, is the factors which the tribunal found relevant in reaching its conclusion, including the fact that there was no clear plan or well-defined intention to exploit the players’ image, the lack of evidence or independent advice to illustrate how the club valued the players’ image at the price which it did, the lack of resources available to the Club to enable them to exploit Giovanni’s image, as well as lack of likelihood that such image rights could be commercially exploited, given they never had been before.^42^ These conclusions, amongst others, provide athletes, clubs and tax advisors a clear illustration of the factors which tax tribunals will take into consideration when assessing the legitimacy of image rights payments. Although it has been argued that “when added to professional guidance, this enables a robust approach to image rights planning under the current legislation and guidance”,^43^ this paper will show that in spite of the useful guidance of the tax tribunal in *Hull City*, the recognition of image rights within taxation without a structured legal framework in any other area of law will continue to facilitate a convoluted system of tax which requires reform.

## The importance of ‘image rights’: the ‘how’ and the ‘why’

The value of a star footballers’ image is irrefutable, meaning commercial sponsors and brands are willing to pay considerable sums of money in order to secure the promotional services of these worldwide, ‘household’ names. To put this into context, David Beckham earned £75 million in endorsements alone in 2014, despite this being his first full year of retirement from professional football.^44^ This strategy of employing athletes to promote goods is one which is difficult to comprehend. For example, why would an individual who does not play basketball, or sport at all, purchase a shoe just because Michael Jordan wears it? The answer to this is articulated by Laddie J in *Irvine v Talksport*^45^;

“the reason large sums are paid for endorsement is because, no matter how irrational it may seem to a lawyer, those in business have reason to believe that the lustre of a famous personality, if attached to goods or services, will enhance the attractiveness of those goods and services to their target market. In this respect, the endorsee is taking the benefit of the attractive force which is the reputation or goodwill of the famous person.”^46^

Given this desire to exploit the celebrity personality and the sizeable commercial value of players’ image rights, both clubs and athletes can benefit from image rights deals. These image rights deals can operate in a number of ways. For example, they can take the form of agreements between employer clubs and their players or endorsement agreements between brands and players themselves. In either instance, the club or brand will generally contract with the players’ image rights company in order to exploit the players’ image in return for a fee. In a club context, the standard employment contract used in the English Premier League and indeed across other leagues and sports means that contracting with their star players’ image rights company is imperative. The contract states that “the Club’s use of the players’ image must not be greater than the average for all first team players”.^47^ The issue with this clause is twofold. Firstly, it prevents the club, in their own brand advertisements being able to continually use the image of their most sought-after individuals. Secondly, it prevents them from consistently using their star players’ image in advertisements featuring their commercial partners, deals which are worth substantial sums (Chevrolet paid £64 million in the current 2019/2020 season to feature on Manchester United’s strip)^48^ and it is unsurprising and logical that these partners want the image the most well-known, commercially attractive players in their branding. As such, it becomes imperative for clubs to seek separate contractual agreements with players’ image rights company in order to allow them to contract beyond the limited capacity which the standard EPL contract allows.

The contractual relationship between player image right companies and clubs can also be tax efficient. For clubs, making image rights payments means they do not have to pay national insurance charges at a rate of 13.8% which they would be liable for if the image right payment was classed as remuneration.^49^ This deduction across a number of marketable first team players potentially amounts to a significant commercial saving. For players, any image rights remuneration paid to their company will not be subject to the 45% income tax charge nor the 2% national insurance contributions.^50^ Instead, the image rights company shall be subject to 19% corporation tax.^51^ Players will be liable for capital gains tax on disposal of their asset (their image) to the company,^52^ whilst they will also be liable for income tax should they receive dividends from the company.^53^ This rate is between 32.5% and 38.1% dependent on the amount of the dividend—again, less than the 45% they would otherwise be liable for.^54^ Thus, for those athletes who are fortunate enough to generate endorsement deals through their image, having an image rights company to exploit this image can be financially beneficial and tax efficient, whilst having the same effect for their employer clubs.^55^

## Image rights in law

*The prevailing issue between footballers, image rights companies and HMRC is that no statutory image right exists in UK law, meaning it is difficult to define exactly what a players’ ‘image right’ constitutes within the realms of taxation*. This confusion has led to the media and politicians’ misconceptions that footballers are ‘gaming’ the system, when the reality is that the system is convoluted and requires reform. The most in point definition of an ‘image right’ in this context was provided in *Proactive Sports Management Ltd v Wayne Rooney and others*,^56^ “the term image rights is used to describe rights that individuals have in their personality, which enables them to control the exploitation of their name or picture”.^57^ These image rights, as established, are worth a substantial amount of capital, but the reality should be reinforced; within the UK legal framework, there is no statutory recognised image right. As such, the current system of taxation forces athletes, clubs and their agents to exploit a right which ultimately does not exist in law. In instances in which celebrities have found their image under threat, they have been forced to circumvent the traditional intellectual property remedies. These have generally provided celebrities with redress but have nevertheless has fallen short of a statutory protected right. For example, In *Campbell v MGN*,^58^ supermodel Naomi Campbell received damages after Mirror Group Newspapers published photographs of her attending Narcotics Anonymous, using a traditional breach of confidence action, as did Michael Douglas and Catherine Zeta-Jones when their wedding photos were published by Hello! Magazine, despite their exclusively contract with rival magazine OK!^59^ These cases raised various concerns in applying a traditional intellectual property remedy to a situation which it was not created for,^60^ such as the protection of private information as opposed to confidential as required by the remedy and protecting privacy for commercial purposes.^61^ Similarly and more importantly for the purposes of this article given it is the remedy HMRC refer to in the capital gains manual (discussed as follows), is the traditional remedy of passing off which has also been utilised by celebrities seeking to protect their image.

The tort of passing off is traditionally concerned with the prevention of undertakings ‘passing off’ their products and services as that of another. Typical cases involve the imitation of a company’s product under a similar name, packaging or slogan, all of which are calculated to cause confusion in the mind of the consumer purchasing the product. The traditional passing off remedy requires three key elements, referred to as the ‘classic trinity’. Firstly, the existence of goodwill in the product or service, ensuring recognition and reputation of the brand. Secondly, there has been a misrepresentation concerning the goods or services. Lastly, the result of the misrepresentation is that there has been damage to the goodwill of the claimant.^62^ This remedy has been extended to cases of false endorsement and false merchandising to protect the celebrity image. In *Irvine*, Formula One driver Eddie Irvine’s image was used by radio station Talksport in an advertisement which gave the impression the athlete had endorsed it.^63^ It was held that the three elements of the trinity were met and that passing off could apply in cases of false endorsement.^64^ Most importantly for tax purposes, however, is the acknowledgement of the changing nature of passing off:

“Not only has the law of passing off expanded over the years, but the commercial environment in which it operates is in a constant state of flux…it is common for famous people to exploit their names and images by way of endorsement. They do it not only in their own field of expertise but, depending on the extent of their fame or notoriety, wider afield also. It is common knowledge that for many sportsmen, for example, income received for endorsing a variety of products and services represents a very substantial part of their total income.”^65^

This acknowledgement that athletes have an image worthy of protection is a positive one, and for tax purposes, illustrates that an athletes’ image is worthy of the payment they receive to their image rights company for their promotional services. However, this acknowledgement falls short of creating an image right. The consequence of this is that when clubs or brands are securing the athletes’ image, there is no clear definition as to what they are paying for. There is no guidance what this ‘image right’ should include (the name, likeness, voice of the athlete) nor is there guidance as to when an athlete’s image has become valuable enough to warrant protection or rather a tax deduction, instead it relies upon whether or not there is ‘goodwill’ present. ‘Goodwill’, a concept which applies far beyond image rights to buying products and services or a business as a going concern is also the means by which HMRC measures and quantifies the value of a footballer’s image. This reliance on passing off continued in the case of *Fenty v Arcadia Group Brands (t/a Topshop)*^66^ also known as *Rihanna v Topshop*, where the traditional remedy was extended to also include cases of false merchandising.

Singer Rihanna raised a passing off action against clothing brand Topshop after they sold a t-shirt featuring her image. Rihanna argued this would lead customers to believe she had endorsed the product.^67^ It was held that Topshop had unlawfully exploited Rihanna’s image and that the classic trinity had been met. In relation to goodwill, the court referred to the star as “world-famous”, highlighting a number of promotional deals Rihanna had become party to with the likes of Gucci and River Island, indicative that she had “ample goodwill to succeed in a passing off action of this kind….the scope of her goodwill was not only as a music artist but also in the world of fashion as a style leader”.^68^ However, in coming to this conclusion, Birss J stated that this was a passing off case and that,

“it is important to state at the outset that this case is not concerned with so called ‘image rights.’ Whatever the position may be elsewhere in the world, and however much various celebrities wish there were, there is today in England no such thing as a free standing general right by a famous person (or anyone else) to control the reproduction of their image.”^69^

This statement not only reinforces that no image right exists within the UK legal framework, but that the only way to ‘measure’ the marketability or value of their image is by assessing the goodwill of the celebrity. This calculation of goodwill is ultimately what HMRC has relied upon in controlling the ‘image right’ of footballers and is ultimately the link between the above cases, HMRC and image rights.

## HMRC and the law of passing off

The HMRC capital gains tax manual states that the only “pure” avenue for the protection of an image right under UK law is through passing off.^70^ There are ultimately difficulties in using a traditional intellectual property remedy to solve a 21^st^ problem, but the complexities of using intellectual property laws for issues which they were not designed for is beyond the scope of this article. Instead, for tax purposes, what is important is that in the absence of an image right, passing off is a valid means for footballers to exploit their image in order to seek a lawful tax deduction—albeit a complicated one, open to exploitation and a source of confusion for agents, footballers and clubs. Is it this complicated system of tax which relies upon a right which does not exist in law that should be ‘grabbing the headlines’, rather than the continued public scrutiny of footballers.

The manual acknowledges the classic trinity discussed above and goes on to discuss the success of *Irvine*, using this as its single source for the proposition that passing off can be used to protect image rights.^71^ As established through the judicial reasoning above, this is correct, but it does not remove the ambiguity surrounding what exactly constitutes an image right. In particular, when players assign their image to the image rights company, the asset they are actually assigning is goodwill. Goodwill is not defined in the relevant legislation (Taxation of Capital Gains Act 1992). It is taken to mean “the benefit and advantage of the good name, reputation and connection of a business. It is the attractive force which brings custom”.^72^ Given the vast amount brands and sponsors are willing to pay for the commercial services of footballers, it can be concluded that generally, footballers do have goodwill in their image, the extent of which will vary depending upon each individual. The existence of this goodwill, however, raises the question as to whether it is capable of assignment for capital gains purposes.

The starting point is that if goodwill is being transferred, an underlying business activity must exist, otherwise it shall be regarded as personal goodwill and thus incapable of assignment. As HMRC explains,

“Goodwill is a personal property and its ownership can be transferred by assignment, but it cannot be assigned ‘in gross’—separate from the business which it relates to. An assignment in gross is invalid and the assignee acquires no rights from such a purported assignment. It may be that an assignment of ‘image rights’ in the context is an assignment in gross of goodwill, if the goodwill is the sportsman’s identity indicia and the business becomes separated from the goodwill. If the business is the sportsman himself and this transaction purports to separate the goodwill in the sportsman and pass it to a company, this is an assignment in gross. On the other hand, if a celebrity has prior to the assignment developed a business based on organised and planned exploitation of their image, for example by endorsements, personal appearances and maybe through contracts for sponsorship, copyright and so on, it is possible that such a business could have established goodwill of some value. If the existing business is transferred with its goodwill as a going concern to the IRC, there is no assignment in gross.”^73^

Thus, if footballers assign their image to an image rights company after one endorsement deal, this would potentially constitute an assignment in gross, dependant on the value of the deal. However, should footballers be able to provide a record of endorsement deals and sponsorship, they can prove to HMRC that the tax deduction is legitimate. Pragmatically, footballers will not decide to transfer their image to a company until it becomes tax efficient, and thus a record of endorsement and a business based on the exploitation of their image will generally be present and relatively straightforward to prove. That being said, it is important to acknowledge that this is not always the case. The very existence of the compliance project is indicative that there are issues surrounding the transfer of goodwill, for example, players stating their image is worth than it is in reality. However, there will always be those who attempt to stretch the boundaries of the law in order to seek a tax advantage, but the confusion surrounding the taxation of footballers’ image rights is ultimately a result of the reliance on passing off which neither defines nor provides guidance as to what exactly image constitutes or includes. Thus, the existence of an image right in one area of law (tax) and not in another is fundamentally to blame for the convoluted system of athlete taxation in relation to image rights in the UK. *This fact is not discussed at any great length nor is it portrayed in this light by the media, who instead scrutinise those footballers under investigation instead of highlighting the intrinsic difficulties faced by athletes, agents and their club in navigating this contentious area of law.*

### Copyright and trademarks

Although HMRC states that passing off is the only ‘pure’ avenue to protect an image right, it is also entirely plausible to argue that the existence of a copyright and/or trademark in one’s image is potentially useful. There is various academic opinion^74^ which disputes the use of copyrights and trademark in protecting the image of a celebrity, given the specifics of the rights and the purposes for which they were originally legislated for. However, given the success of breach of confidence and passing off in protecting the image of a celebrity, it is not outside the realms of possibility that the scope of copyright and trademark law could be extended to similarly protect an athlete’s image. This, in turn, would allow satisfying HMRC that the assignment of the image is not an assignment in gross.

Copyright is legislated for by the Copyright, Designs and Patents Act (1998), which defines work worthy of copyright protection as an ‘original literary, dramatic, musical or artistic work’.^75^ As such, it has been argued that s.1 provides a basis for the protection of a celebrity’s image in the absence of a statutory image right.^76^ In spite of this, the absence of recent case law and the opinion of the Whitford Committee in 1977 have created ambiguity as to the applicability of copyright law.

The protection of the celebrity persona through copyright law was first rejected by the Whitford Committee, who explicitly dismissed efforts to establish a “character right in fictitious figures”.^77^ It is worth noting that this is potential to the detriment of athletes, who potentially may want to create copyright in the “fictitious figure” whom they are on the field of play. For example, an athlete may act in a certain way whilst participating in their sport, portraying certain characteristics such as bravery or strength and want to protect copyright in that image, rather than copyrighting the “figure” they are in everyday life.

Generally, the limited and dated case law rejects the use of copyright in the protection of the celebrity image. In *Merchandising Corporation of America Inc. and Others v Harpbond Ltd and Others*,^78^ the plaintiff argued for the existence of copyright in his “new look” which consisted of an extravagant hairstyle and matching make-up. The court held no copyright could exist in distinctive hair or make-up nor could it exist in an individuals’ “new image”, by virtue of the fact that the image could not be “fixed” to anything. In fact, the court went so far as to say “a painting must be on a surface. If there were a painting in this case it must be the make-up marks plus the second plaintiff’s face. If the marks were taken off the face, there could not be a painting”,^79^ thus explicitly rejecting it on the basis of the element of fixation.^80^

However, there must be an acknowledgement that the Whitford Committee took place in 1977 and *Harpbond* in 1983. Given the success of breach of confidence and passing off in protecting image, coupled with the wide scope of s1 and the continued absence of a statutory image right, the protection of a celebrity’s image through copyright may prove possible, should one attempt to utilise it.

In relation to trademarks, the over-riding issue in protecting the image of a celebrity through this mechanism is the contrast in functions between the two. A trademark is defined under the Trademark Act (1994) which states that a trademark is any sign capable of “distinguishing goods or services from one undertaking from those of other undertakings”.^81^ The primary function of a trademark is to serve as an indication of origin or source, demonstrated by case law such as *L’Oreal v Bellure*^82^ in which it was stated that “the essential function of the trademark…is to guarantee to consumers the origin of the goods or services”.^83^ Conversely, “in cases where goods are connected to a famous image, the personality of the celebrity is considered more important than the indication of origin, thus rendering trademark law inapplicable”.^84^ For example, a consumer who purchases a Cristiano Ronaldo poster does so on the basis of Ronaldo himself; it is his image as a famous footballer which the consumer is concerned with, not the source of the product. Put simply, “trade origin is not a celebrity’s concern; a personality mark is about the glitter and glamour that the association with a celebrity confers on the goods on which it is used”.^85^

These contrasting functions and thee incompatibility of trademark law and image rights have not stopped attempts made by celebrities to utilise trademark law to protect their persona.

In 1997, the Memorial Fund attempted to trademark 52 different pictures of the late Princess Diana, all of which were rejected on the basis that her face was too well known to be limited as the badge of one individual supplier.^86^ This, in theory, highlights a potential issue for celebrities who which to trademark their image, in that they may lose distinctiveness in their image through continued use, therefore no longer warranting the protection of trademark law. However, the reality is that for tax purposes, the ability to trademark their image in the first place is what is important, as it provides evidence to HMRC that the assignment of their image to an image rights company is not one in gross. In spite of Diana, there have been successful trademark applications by athletes in their quest to protect their image.

Formula One driver Damon Hill successfully trademarked the image of his eyes through his visor on his racing helmet.^87^ More recently, England and Manchester United footballer Jesse Lingard also applied successfully for four trademarks before appearing in the 2018 World Cup; three featuring his nickname “JLingz” and another image of his goal celebration.^88^ This is particularly useful for athletes who own image rights companies and the potential importance of this should not be underestimated. As above, the issues regarding the use of trademarks and copyright and traditional intellectual property remedies generally to protect the image of an athlete are beyond the scope of this article. As far as athletes and their agents are concerned, if through the Intellectual Property Office, they are able to trademark their image under s1 or obtain copyright under the CDPA, the above legal issues surrounding applying and perhaps stretching the boundaries of these traditional intellectual property laws in order to protect the ‘celebrity image’ are not of relevance to them. In any case, until a copyright or trademark obtained by an athlete is infringed, the question remains as to how the courts or the legislature will deal with the issues outlined above. It is not inconceivable that the courts may interpret trademark and copyright law in a similar manner to the law of passing off to allow celebrity’s legal protection of their image through this traditional remedy.

What is important for the purposes of taxation is that the ability to register a trademark or if an image gives rise to copyright protection then this is simply a useful weapon in proving to HMRC that they have an image worth protecting and that there is no assignment in gross when transferring their image to the image right company. The ability to copyright or trademark their image in the first instance provides evidence to HMRC that they possess the requisite goodwill in their image to warrant protection or rather exploitation and ultimately a tax reduction. This circumvention of the traditional remedies is necessary due to the continued non-existence of an image right. Until this is rectified, the system of tax in the UK in relation to footballers and image rights will remain convoluted at best, and at worst, open to abuse.

## Concluding thoughts and moving forward

Reports on possible tax avoidance by footballers have drawn the attention of the media for a number of years. The Compliance Project and its additional revenue illustrate that this is a continuing problem and one which HMRC is pursuing. However, in the midst of the COVID-19 pandemic, the taxation of athletes has once again been brought to the attention of the media and the public, through the scrutiny of politicians. Given the ‘#playerstogether’ initiative, the money donated to the NHS by professional footballers, and the fact that athletes have potentially lost a year of their careers, perhaps rather than ‘calling out’ individuals for tax avoidance, it should be acknowledged (as it was by the PFA) that athletes contribute a significant amount of tax through the PAYE system. Thus, instead of criticising athletes for their tax affairs, there should be an acknowledgement that the current system is convoluted, confusing for all, and ultimately, requires reform.

In summary, the predominant issue regarding athletes using an image right company to structure their tax affairs in order to facilitate a lower tax rate remains. It is the fact that a ‘right’ which does not exist one area in law is protected and exploited in another. This fact is not explored nor published by the media. This article has illustrated that the traditional intellectual property remedies such as breach of confidence and trademarks have protected the image of celebrities. However, in the context of taxation, HMRC relies solely on the remedy of passing off and the concept of goodwill to quantify or justify the transfer of an athletes’ image to an image right company (although that is not to say that in the continued absence of a statutory image right that copyright or other remedies may not be useful). The issue with using passing off is twofold: again, athletes are exploiting a right which does not exist in law and there is no guidance as to when an athlete’s goodwill is transferable, other than a paragraph in the Capital Gains Tax manual which discusses assignment in gross. This, however, is not the fault of athletes, clubs or their agents. That is not to say that there are not those who push the boundaries of the law, and the Football Compliance Project is evidence of that. *However, suffice to say that the system of tax in relation to athlete image right is convoluted and ultimately needs reform, either through the creation of a statutory image right or by providing clear and transparent HMRC guidance which does not depend on a right which does not exist within the UK legal framework*. The introduction of reform will be far from straightforward but is ultimately required. Guidance could be taken from the Guernsey Image rights register, which is the first of its kind and allows celebrities to register their image for the purposes of protection and exploitation. Such a scheme, coupled with a system of tax akin to that used in Canada which allows athletes to make tax savings in a clear and transparent manner,^89^ would be a positive and much needed step towards improving the system of taxation in relation to athletes’ image rights in the UK. In any case, however, this reform will not be successful without co-operation between the various interested parties: the legislator, politicians and the football industry.

## Footnotes

1 BBC News, Lora Jones, Daniele Palumbo, David Brown, ‘Coronavirus: a visual guide to the economic impact’ (27th April 2020). https://www.bbc.co.uk/news/business-51706225. Accessed 27th April 2020.

2 Ibid.

3 BBC Sport, ‘Tottenham Hotspur reverse furlough decision for non-playing staff’ (13th April 2020). https://www.bbc.co.uk/sport/football/52271235. Accessed 27th April 2020.

4 The Independent, Lawrence Ostlere, ‘Coronavirus: Jamie Carragher was ‘angry’ and ‘embarrassed’ by Liverpool’s furlough plan’ (7th April 2020). https://www.independent.co.uk/sport/football/premier-league/jamie-carragher-liverpool-fc-coronavirus-furlough-latest-news-a9452056.html. Accessed 27th April 2020.

5 The Independent, Jack Rathborn, ‘Piers Morgan brands Liverpool as ‘astonishingly stupid’ for furloughing staff’ (6th April 2020). https://www.independent.co.uk/sport/football/premier-league/piers-morgan-liverpool-furloughing-staff-good-morning-britain-video-a9449326.html. Accessed 27th April 2020.

6 The Guardian, Andy Hunter, ‘Liverpool announce £42m pre-tax profit despite record £223m spend on players’ (27th February 2020). https://www.theguardian.com/football/2020/feb/27/liverpool-42m-pre-tax-profit-record-223m-spend-on-players-financial-year-2018-19. Accessed 27th April 2020.

7 The Independent, Jack De Menezes, ‘Coronavirus: Matt Hancock tells Premier League players to ‘play their part’ and take pay cuts’ (2nd April 2020). https://www.independent.co.uk/sport/football/premier-league/coronavirus-matt-hancock-premier-league-players-pay-cut-wages-health-secretary-nhs-workers-died-a9444041.html. Accessed 14th April 2020.

8 The Independent, Jack De Menezes, ‘Coronavirus, Sadiq Khan criticises Premier League footballers for not taking wage reductions’ (1st April 2020). https://www.independent.co.uk/sport/football/premier-league/coronavirus-premier-league-sadiq-khan-wage-cuts-furlough-spurs-newcastle-a9440226.html. Accessed 27th April 2020.

9 BBC Sport, ‘Coronavirus: Wayne Rooney says footballers treated as guinea pigs’ (15th March 2020). https://www.bbc.co.uk/sport/football/51896488. Accessed 27th April 2020.

10 The Guardian, ‘Wayne Rooney leads footballers’ backlash against pay cut criticism’ (5th April 2020). https://www.theguardian.com/football/2020/apr/05/wayne-rooney-leads-footballers-backlash-against-government-and-premier-league-lineker-neville. Accessed 27th April 2020.

11 Professional Footballers Association, ‘PFA statement on behalf of Premier League players’ (4th April 2020). https://www.thepfa.com/news/2020/4/4/pfa-statement-on-behalf-of-premier-league-players. Accessed 14th April 2020.

12 BBC Sport, ‘NHS: Premier League players’ initiative to generate and distribute funds’ (8th April 2020). https://www.bbc.co.uk/sport/football/52219771. Accessed 27th April 2020.

13 BBC Sport, ‘HMRC investigations into footballer tax affairs reach new level’ (3rd March 2020). https://www.bbc.co.uk/sport/football/51698150. Accessed 14th April 2020.

14 Ibid.

15 Ibid.

16 For a wider discussion of this point in relation to stamp duty, see Project Blue Limited v Commissioners for HMRC [2018] UKSC 30.

17 Judith Freedman, Current Legal Problems, ‘The Tax Avoidance Culture: Who is Responsible? Governmental Influences and Corporate Social Responsibility’ (2006), Volume 59, page 2.

18 (2000) S.T.C. (S.C.D) 443.

19 Freddie Huxtable, European Professional Football Leagues Newsletter (#5) ‘Image Rights in the UK’. https://europeanleagues.com/wp-content/uploads/Article-5-Image-rights-UK-2-2.pdf. Accessed 27th April 2020.

20 Corinna Coors, International Sports Law Journal, ‘Are sports image rights assets? A legal, economic and tax perspective’ Page 5.

21 (2000) S.T.C. (S.C.D.) 443, [2].

22 Ibid [74].

23 Ibid [79].

24 Ibid [80].

25 Ibid [90]–[95].

26 Ibid [100].

27 Judith Freedman, Current Legal Problems, ‘The Tax Avoidance Culture: Who is Responsible? Governmental Influences and Corporate Social Responsibility’ (2006), Volume 59, page 2.

28 Daniel Geey, ‘Five top tips to understand football image rights deals’ (26th October 2016). https://www.danielgeey.com/post/five-top-tips-to-understand-football-image-rights-deals/. Accessed 27th April 2020.

29 Corinna Coors, International Sports Law Journal, ‘Are sports image rights assets? A legal, economic and tax perspective’ Page 5.

30 Ibid.

31 Kevin Offer, Tax Journal, ‘Taxation of Image Rights’ (21^st^ March 2018), page 1.

32 Daniel Geey, ‘Five top tips to understand football image rights deals’ (26th October 2016). https://www.danielgeey.com/post/five-top-tips-to-understand-football-image-rights-deals/. Accessed 27th April 2020.

33 Ibid.

34 [2017] UKSC 45.

35 Ibid [39].

36 Kevin Otter, Tax Journal, ‘Taxation of Image Rights’ (21st March 2018), page 1.

37 [2019] UKFTT 227.

38 It is noteworthy that the rulings of the Special Commissioners or the Tax Tribunals are not binding and do not set judicial precedent, but are a useful guide to the law.

39 Ibid [3].

40 Ibid [5].

41 Ibid [128].

42 Ibid.

43 Jon Elphick, Tax Adviser, ‘Image Rights’ (1st September 2019). https://www.taxadvisermagazine.com/article/image-rights. Accessed 4th November 2020.

44 Forbes, Kurt Badenhausen, ‘David Beckham banks biggest ever year with earnings of $75 million’ (11th March 2015). https://www.forbes.com/sites/kurtbadenhausen/2015/03/11/david-beckham-banks-his-bigger-year-ever-with-earnings-of-75-million/#52b6ce5e5775. Accessed 27th April 2020.

45 (2002) W.L.R. 2355.

46 Ibid [2368].

47 Daniel Geey, Pete Hackleton, ‘Image Rights in UK Football Explained’. https://www.danielgeey.com/post/image-rights-in-uk-football-explained/. Accessed 27th April 2020.

48 Statista, ‘Value of jersey kit sponsorship in the Barclays Premier League in 2019/2020’. https://www.statista.com/statistics/254513/value-of-jersey-kit-sponsorships-in-the-barclays-premier-league-by-club/. Accessed 10th February 2020.

49 HMRC Guidance Notes, “National Insurance Rates and Categories”. https://www.gov.uk/national-insurance-rates-letters. Accessed 14th April 2020.

50 HMRC Guidance Notes (1st April 2017) Rates and Allowances: National Insurance Contributions. https://www.gov.uk/government/publications/rates-and-allowances-national-insurance-contributions/rates-and-allowances-national-insurance-contributions. Accessed 14th April 2020.

51 HMRC Guidance Notes (1st April 2017) “Rates and Allowances: Corporation Tax”, https://www.gov.uk/government/publications/rates-and-allowances-corporation-tax/rates-and-allowances-corporation-tax. Accessed 14th April 2020.

52 HMRC Guidance Notes (26th May 2017) “Capital Gains Manual”. https://www.gov.uk/hmrc-internal-manuals/capital-gains-manual. Accessed 14th April 2020.

53 Kevin Offer, Tax Journal, ‘Taxation of Image Rights’ (21st March 2018), page 2.

54 HMRC Guidance Notes, “Tax On Dividends”, https://www.gov.uk/tax-on-dividends. Accessed 14th April 2020.

55 For further discussion on taxing businesses specifically, see Stuart Adam, Helen Miller, Institute for Fiscal Studies, ‘Principles and practice of taxing small business’ (December 2019), https://www.ifs.org.uk/uploads/WP201931-Principles-and-practice-of-taxing-small-business.pdf. Accessed 4th November 2020.

56 (2001) EWHC Civ. Div. 1444.

57 Ibid [1].

58 (2004) UKHL 22.

59 (2007) UKHL 21.

60 For example, see Catherine Walsh, European Intellectual Property Law Review, ‘Are personality rights finally on the UK agenda?” (2013) 253, 237.

61 For further analysis, see Hazel Carty, Intellectual Property Quarterly, ‘An analysis of the modern action for breach of commercial confidence’ (2008) 416, 466.

62 See for example, Consorzio del Proscuitto di Parma v Marks and Spencer Plc (1991) R.P.C. 35, Reckitt and Coleman Products Inc v Borden (1990) 1 All E.R. 873 and Erven Warnink B.V. v J. Townend and Sons (Hull) Ltd. (1979) All E.R. 927.

63 (2002) 1 W.L.R. 2355 [4]–[8].

64 Ibid [56], [68], [74].

65 Ibid [39].

66 (2013) EWHC 2310 (Ch); (2014) E.C.D.R. 3; (2015) 1 W.L.R. 3291.

67 (2013) EWHC 2310 (Ch) [47].

68 Ibid [45]–[46].

69 Ibid [2].

70 Capital Gains Manual, ‘Intellectual Property Rights: image rights: the Law of Passing-Off’. https://www.gov.uk/hmrc-internal-manuals/capital-gains-manual/cg68410. Accessed 14th April 2020.

71 (2002) 1 W.L.R. 2355.

72 Capital Gains Manual, ‘Goodwill: meaning of goodwill’. https://www.gov.uk/hmrc-internal-manuals/capital-gains-manual/cg68010. Accessed 15th April 2020. Definition taken from *Inland Revenue Commissioners v Muller & Co. Margarine Ltd* (1901) AC 217, [235].

73 Capital Gains Tax Manual, ‘Intellectual Property Rights: assignment of image rights.’ https://www.gov.uk/hmrc-internal-manuals/capital-gains-manual/cg68420. Accessed 27th April 2020.

74 For example, see Savan Bains, Entertainment Law Review, ‘Personality Rights: should the UK grant celebrities a proprietary right in their personality? Part 1’ (2007) 164.

75 Copyright, Designs and Patents Act (1988) s.1.

76 Ibid at 166.

77 Jan Klink, Intellectual Property Quarterly, 50 years of publicity rights in the United States and the never ending hassle with intellectual property and personality rights in Europe’ (2003) 363, 370.

78 (1983) F.S.R. 32.

79 Ibid [46].

80 See also Exxon Corp v Exxon Insurance Consultants International Ltd (1981) 1 W.L.R. 64, (1981) F.S.R. 238.

81 Trademark Act (1994) s.1(b).

82 Case C-497/07.

83 Ibid [58].

84 Savan Bains, Entertainment Law Review, ‘Personality Rights: should the UK grant celebrities a proprietary right in their personality? Part 1’ (2007) 164, 165.

85 Catherine Walsh, European Intellectual Property Law Review, ‘Are Personality Rights Finally on the UK Agenda?’ (2013) 253, 256.

86 BBC News, ‘UK Diana fund loses trademark battle’ (1999). http://news.bbc.co.uk/1/hi/uk/272380.stm. Accessed 15th April 2020.

87 the Independent, ‘Lumley finds bus ad absolutely outrageous’ (1997). https://www.independent.co.uk/news/lumley-finds-bus-ad-absolutely-outrageous-1291645.html. Accessed 15th April 2020.

88 Joe Hall, City A.M, ‘World Cup 2018: England’s Jesse Lingard has trademarked his JLingz celebration—and he’s done so at the perfect time’ (17th June 2018). https://www.cityam.com/world-cup-2018-englands-jesse-lingard-has-trademarked-his/. Accessed 15th April 2020.

89 For further information on the Canadian Tax System, see Sarah Carrick, “Athlete Image Rights and the potential consequences in the UK and Canada” (2019) available via the University of Stirling.
